# Transforming Health Care in Remote Communities: report on an international conference

**DOI:** 10.1186/s12919-016-0006-0

**Published:** 2016-08-10

**Authors:** T. Kue Young, Susan Chatwood, James Ford, Gwen Healey, Michael Jong, Josée Lavoie, Mason White

**Affiliations:** 1 School of Public Health, University of Alberta, Edmonton, AB Canada; 2 Institute for Circumpolar Health Research, Yellowknife, NT Canada; 3 Dalla Lana School of Public Health, University of Toronto, Toronto, Canada; 4 Department of Geography, McGill University, Montréal, Canada; 5 Qaujigiartiit Health Research Centre, Iqaluit, NT Canada; 6 Northern Ontario School of Medicine, Sudbury, Canada; 7 Labrador-Grenfell Regional Health Authority, Happy Valley-Goose Bay, Newfoundland and Labrador Canada; 8 Discipline of Family Medicine, Memorial University of Newfoundland, St. John’s, Newfoundland and Labrador Canada; 9 Department of Community Health Sciences, Centre for Aboriginal Health Research, Faculty of Health Sciences, University of Manitoba, Winnipeg, Canada; 10 John H. Daniels Faculty of Architecture, Landscape and Design, University of Toronto, Toronto, Canada

## Abstract

An international conference titled “Transforming Health Care in Remote Communities” was held at the Chateau Lacombe Hotel in Edmonton, Canada, April 28–30, 2016. The event was organized by the University of Alberta’s School of Public Health, in partnership with the Institute for Circumpolar Health Research in Yellowknife, Northwest Territories, and the Qaujigiartiit Health Research Centre in Iqaluit, Nunavut.

There were 150 registrants from 7 countries: Canada (7 provinces and 3 territories), United States, Denmark, Iceland, Norway, Sweden, and Australia. They included representatives of academic institutions, health care agencies, government ministries, community organizations, and private industry.

The Conference focused on developing solutions to address health care in remote regions. It enabled new networks to be established and existing ones consolidated.

## Introduction

Planning, organizing and delivering health care for remote, sparsely populated communities pose serious financial, logistical, technical, and human resource challenges. In response to these challenges, northern Canada, as well as its neighbours across the circumpolar North - Alaska, Greenland, the Nordic countries and Russia - have evolved vastly different health care systems and policies. Despite climatic differences, Australia also has sparsely populated regions with a substantial Indigenous population and many similar health care issues.

The timing was right for a forum which brought together Canadian and international policy-makers, administrators, clinicians, practitioners, community partners, researchers, students, and industry representatives to synthesize what have been learnt to date, share and disseminate the results, and to plan and explore continuing and future collaborations.

### Conference objectives

The conference addressed several key questions:

- What are the key challenges and emerging issues?
- What solutions have been tried and do they work?
- What can we learn from other jurisdictions?
- What can we do differently to improve health and health care?

### Opening remarks

The Conference opened with a prayer and song by Be’sha Blondin, traditional Sahtu Dene elder from Tulita, Northwest Territories. She put health care in the context of Indigenous spiritual and cultural values and tasked the audience to work towards the betterment of Indigenous and non-Indigenous people living in remote communities.

Bringing greetings on behalf of the University of Alberta, Roger Epp, Professor and Director of UAlberta North, pointed out that “*one person’s ‘remote’ is another’s epicenter*”. The use of the term ‘remote’ unconsciously exposes the speaker’s perspective, which is that of an outsider, often an external expert attempting to impose solutions on communities. As emphasized by Kue Young, Dean of the School of Public Health, it was the intent of this conference to engage participants collaboratively to identify unresolved problems and share best practices.

The presence of senior policy and decision makers enhanced the value and relevance of the conference. Glen Abernethy, Minister of Health and Social Services, Government of the Northwest Territories, highlighted the challenges faced by his government. He spoke about the policies and programs he and his staff had instituted to make health care more accessible, effective, and efficient. “*Our vision is a single system with a coordinated approach to achieve best health, best care, for a better future for our people*”, said Debbie DeLancey, deputy minister, who provided further details on transformative changes in the Northwest Territories. Rosemary Keenainak, assistant deputy minister, Nunavut Department of Health, presented the perspective from her territory and communities. She highlighted the importance of science and traditional knowledge in understanding and improving community wellness.

### Plenary addresses

There were five plenary addresses by distinguished international guests, who discussed different aspects of remote health care:

- Peter Bjerregaard, Professor of Arctic Medicine, Centre for Health Research in Greenland, National Institute of Public Health, Copenhagen, Denmark, and University of Greenland, Nuuk, Greenland 1
- Ivar Mendez, Professor and Chair, Department of Surgery, University of Saskatchewan, Saskatoon, Canada 2
- Vanessa Hiratsuka, Senior Researcher, Southcentral Foundation, Anchorage, Alaska, United States 3
- Árún K. Sigurðardóttir, Professor and Dean, Faculty of Health Sciences, University of Akureyri, Iceland 4
- Graeme Maguire, Professor and Head, Clinical Research, Baker IDI Heart and Diabetes Institute, Melbourne and Alice Springs, Australia 5

These addresses were spread out throughout the Conference and their contents are discussed in the rest of this report under the relevant themes. Serving as moderators for these plenary sessions were the presidents of two important organizations devoted to remote health care: Christina Viskum Lytken Larsen of the Circumpolar Health Research Network, and Deirdre Jackman of the Canadian Association for Rural and Remote Nursing.

### Presentation themes and topics

The Conference was organized around seven themes: Policies, People, Places, Links, Tools, Costs, and Data. In addition to the 5 plenary addresses, there were 41 oral presentations, 3 panel discussions, and 17 posters.

#### Policies

Policies determine and shape health systems. Bjerregaard described the health care reform in Greenland, which was implemented in 2011. It replaced 16 small district hospitals with 5 large regional ones. A major motivation for the reorganization was to improve physician recruitment, but it remained a continuing problem. There were mixed reviews regarding the other changes. A similar centralizing approach has recently been adopted in the NWT, whereby the current 8 regional health authorities serving 45,000 people were to be consolidated into three.

Lavoie and Kornelsen 6 compared the designs of health services for Indigenous people in different circumpolar regions, where there are a range of provisions from a completely separate and parallel system, such as the Alaska Native tribal system (described by Hiratsuka in her plenary) to the Nordic countries and Russia where the proportion of Indigenous people is small and few provisions exist to ensure that services are culturally appropriate. In jurisdictions where Indigenous people are the majority, as in Nunavut and Greenland, there is no distinction between the territorial health system and an Inuit health system.

**Fig. 1:**
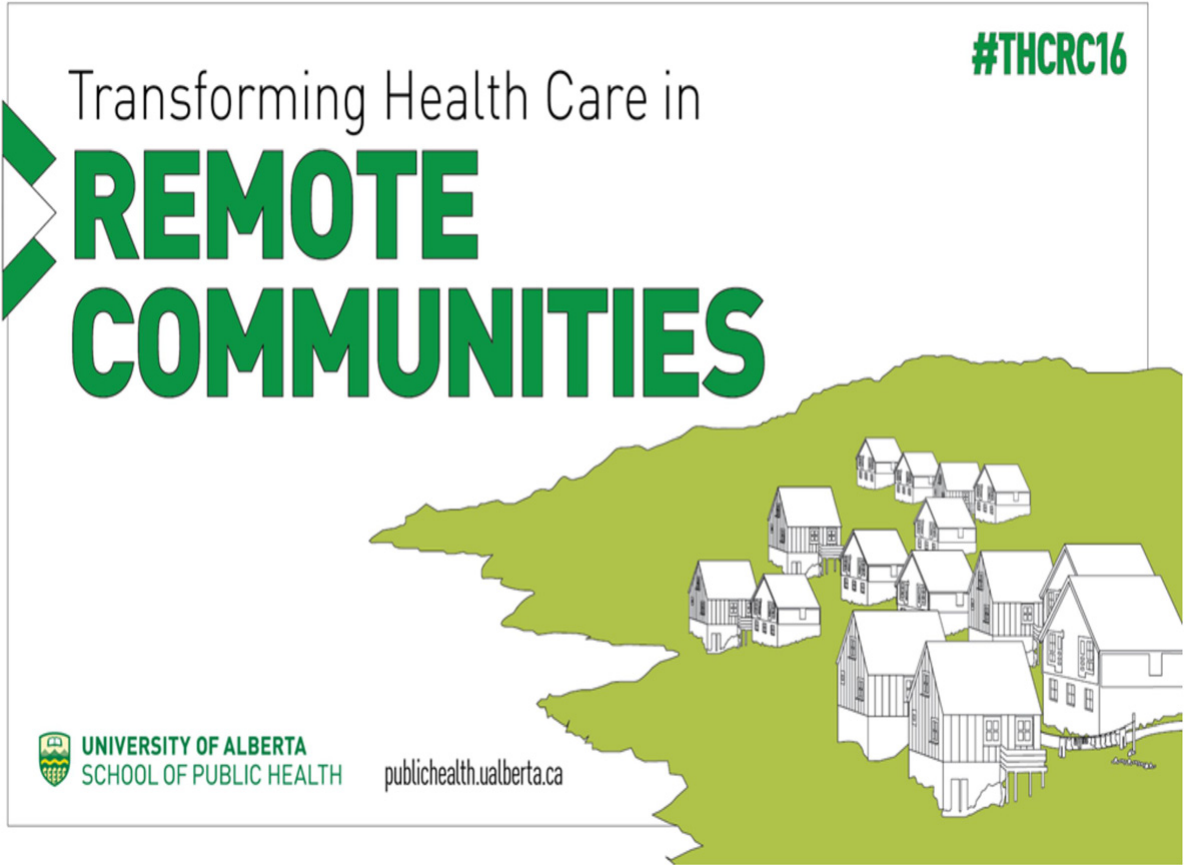
Poster of the International Conference on Transforming Health Care in Remote Communities

Although the Conference focused on primary health care, it also recognized the importance of going “upstream” in understanding what influences the health of remote communities (Fig. 1). Chatwood 7 highlighted how health systems can respond to the social determinants of health. Driscoll and Ray 8 presented their systematic review and identified the most important social and environmental determinants of mortality in circumpolar regions. Johnson 9 proposed viewing remote health from an equity lens. The concept of “vulnerable population” was explored by Maguire ^5^ in the Australian context. While a useful concept in ensuring equity among all segments of the population, it also runs the risk of further stigmatizing an already disadvantaged group.

The policy-practice gap was examined by Romain 10, using the delivery of pharmacy services in Nunavut as a case study. Northern patients with mental health problems are at risk of falling between the justice and heath care systems, as Drossos 11 examined the various mental health acts in northern Canada and pointed out their lack of accommodation for Indigenous cultural values. Kandola and Andrews 12 demonstrated the important role played by municipal and local governments in initiating and implementing healthy public policies.

#### People

“People” are central to any health system, comprising the workforce and the people that it serves, which is the entire population, whether they use the services provided or not. Hiratsuka ^3^ informed the audience that in the Alaska Native tribal health system, the term “customer” is used in place of “patient”, which encourages a non-hierarchical relationship.

Rondeau 13 outlined the problems in health human resources planning. The solutions can be approached from both the supply and demand side. The former can be achieved by recruitment and retention efforts, efficiency in medical travel, and professional substitution, and the latter by reducing service utilization through prevention and community wellness strategies.

In remote communities, front line health workers such as nurses and family physicians need to work collaboratively. MacDonald and Berry 14 discussed challenges such as scope of practice constraints, high staff turnover, role ambiguity, and lack of training in inter-professional collaboration. Jackman 15 and colleagues described an innovative inter-professional preceptorship program at the University of Alberta whereby medical and nursing students were trained together in rural settings, and learned to communicate effectively and respect each other’s professional roles. Urban-based educational institutions have the responsibility to reach out to and recruit students from remote communities, a case made by D’Hont 16, a student from the Northwest Territories.

Nurses are the backbone of the health system, and a panel “Learning Where You Live” organized by Exner-Pirot 17 presented different models of educating nurses for remote and rural practice in Norway, Canada, Iceland and Russia. Moffit 18 presented data on Indigenous nurses from a national survey of nurses working in rural and remote communities in Canada.

From farther afield, Yue presented her practicum project on the extent of trust caregivers had in their community health workers (CHWs) in rural Uganda 19. CHWs are also used extensively in Alaska, where they are called community health aides, and in some smaller communities in northern Canada where there are no resident nurses.

“People” also refers to building relationships with communities and other partners. Montesanti 20 developed an international network on chronic disease prevention involving researchers, government officials and representatives of Indigenous communities. Heffernan, Komarnisky and colleagues 21 described how they built a team across three western Canadian provinces in crafting an intervention program to control tuberculosis. Jardine 22 identified the critical role of trust between academic researchers and Indigenous communities in research addressing health inequities. Geary 23 organized a panel of researchers and community members (from Aklavik, Ulukhaktok and Fort McPherson in the Northwest Territories) and shared their experience with the CANHelp project initiated in response to community concerns with stomach cancer.

#### Places

“Places” refer not just to where health care is delivered and used, but also the broader physical and social environment, from home, workplace, neighbourhood, community, to the “backcountry,” wherever human activities occur and contribute to health and wellbeing.

Healey and her team 24 introduced Sangilirviuksaq (“a place to gather strength”), a healing model incorporating Inuit views of the relationship between wellness and the land in the design of health care spaces in Nunavut. Environmentally and culturally sensitive architectural designs produced by winners of a student competition from this project were illustrated in the poster by White 25.

Larcombe 26 reviewed the diverse health impact of housing conditions on health among the Dene in northern Manitoba and reported on a partnership with the community in the planning and design of healthy and sustainable housing systems. Morar and Caron-Ray 27 drew attention to the importance of public places to promote social interactions and how social infrastructure had changed as Inuit moved from traditional settlement patterns to contemporary townsites.

For those engaged in the wage economy, a substantial portion of their daily lives are spent in the workplace, which presents an opportunity to encourage healthy behaviours, according to Anderson and Kandola 28.

Inuit communities in northern Canada are facing severe environmental stresses as a result of climate change and resource extraction industries, with both direct and indirect impacts on health. Sawatzky 29 described a community-based surveillance system to monitor environmental change in Labrador. A visible impact of environmental change described by Clark 30 is in the increasing number of land-based injuries and search-and-rescue incidents, which has implications for emergency preparedness and health system response.

The theme of connection to the land in fostering healing and reclaiming health was explored by Young and Blondin 31 and MacKenzie 32.

#### Links

The transportation and telecommunication systems provide the critical links between remote communities and regional centres where more complex care is provided. Such links are no less important in Iceland, where the concept of “remote” is very different from that of Canada in terms of actual distance and road access.

Aeromedical evacuations (or “medevacs”) consume a substantial proportion of the health care budgets of northern jurisdictions. A panel discussion was organized by Macdonald 33, with panelists from Nunavut, Northwest Territories, Labrador and Greenland. MacDonald estimated the number and rate of medevacs, with substantial variation across regions as summarized in Table 1.

**Table 1:** Estimated number and rate of aeromedical evacuations by territory

Region	Number per year	Population	Rate (per 100,000)
Nunavut	1800	32,000	5,625
Northwest Territories	1100	44,000	2,500
Labrador	210	15,000	1,400
Greenland	354	55,000	644

Understanding the reasons for such regional differences is key to improving the effectiveness and efficiency of remote health care. Innovative ideas such as using balloons instead of fixed-wings aircrafts have been proposed. White 34 explored the concept of using airport hangars as nodes in a regional network of health care facilities suited to Nunavut’s geography and culture.

The transfer of critically ill patients begins at the point of first contact, but there is little formal training of community-based paramedical technicians (“paramedics”). Orkin 35 reported on a demonstration project in training paramedics in remote First Nations communities in northwestern Ontario. He emphasized that the health system should regard the health centre or nursing station not as an inadequate hospital, but as an ambulance without wheels, where improvement in equipment and the skills of providers could improve equity in access and health outcomes. In northeastern Ontario, Sherman 36 described the integration of paramedics in non-emergency health care such as assessment and referral, home visits, and wellness checks, to fully utilize their skills during “down” time when there are no emergencies.

#### Tools

In his plenary address, Mendez provided a state-of-the-art review of remote presence technology and emphasized how it can be used to redress disparities in access to specialist services. Mendez is a pioneer in the use of remote control robots to perform surgery. Jong 37 reviewed the progress in the use of telehealth in Labrador over the past 15 years, culminating in the deployment of “Rosie the robot” in one remote community, which he demonstrated live to the delight of the audience. Kawaguchi 38 presented a systematic review of tele-ophthalmology in the diagnosis of macular degeneration and diabetic retinopathy. From the conference, Edin-Liljegren 39 took the audience to her “virtual health room” in northern Sweden where patients can be managed by providers who are far away. In northern Alberta, Ross 40 evaluated a telehealth project in partnership with First Nations and found that the technology was well accepted by the community.

Telehealth technologies can be used not just for patient care, but also in the training of health workers. Butler and Exner-Pirot described their project in the remote training of nurses in Yakutsk, Russia from their base in Saskatoon, Canada. Teleconferencing also plays a role in the court system in Nunavut in the psychiatric assessment of offenders, enabling remote access to experts, according to Ferrazzi 41.

Manca 42 presented the Building on Existing Tools To ImprovE Chronic Disease Prevention and Screening in Primary Care (or BETTER) trial which utilized web-based and electronic medical record-enabled resources to support prevention practitioners and facilitators.

Joschko 43 described an e-consult service, a secure online platform allowing asynchronous communication between family physicians and specialists and exchanges of clinical information. About two-thirds of consultations were resolved without a need to see the specialist, resulting in savings in time and costs associated with waiting for and travel to appointments.

Ryan 44 reported on the steps involved in developing a phone app for expectant mothers called the Yukon Baby App, making use of volunteer programmers from Hacking Health North.

McCready 45 described how communication between on-call physicians in the regional hospital and nurses in the communities was improved by replacing a hand-written “community call form” transmitted by fax with a fillable pdf form transmitted by email.

Telus Health 46 developed a home health monitoring protocol for heart failure. Tested in two British Columbia health authorities, it involved a tablet computer connected to peripheral devices that measure blood pressure, pulse and weight. The data were transmitted to nurses in designated locations where they were monitored and action taken. The trial demonstrated reduction in heath care utilization and can be adapted for other chronic conditions.

#### Costs

With their widely dispersed population, health care expenditures on a per capita basis are considerably higher in northern Canada than elsewhere in the country, and indeed are among the highest in the world. Young 47 attempted to address whether there was value-for-money in terms of health outcomes. In Canada, health care costs are borne by multiple levels of government. Galloway 48 studied the funding arrangements between the federal government and Yukon First Nations and discovered wide discrepancy in program access and funding. Investment in “conventional” centralized water and sanitation infrastructure in remote Arctic communities is costly, whereas sustainable, safe and less expensive alternatives have been developed and could be implemented, according to Ashbolt and Shoults 49, based on their studies in Alaska.

#### Data

Health systems generate data, and data in turn provide evidence for improving health systems. Utilization data can be captured from the entire population through administrative databases, or alternatively using sentinel surveillance, whereby a sample of facilities or providers report electronically their health care data to a central data repository. Manca 50 described the Canadian Primary Care Sentinel Surveillance Network which covers >1000 primary care sites across Canada, including the Northwest Territories. Not only does the network generate data on usage patterns and disease prevalence, it can also be used as a tool for quality improvement.

Moffit 51 described how geographic information system (GIS) maps were used to visualize the spatial pattern of intimate partner violence in the Northwest Territories, which can serve as a communication and knowledge mobilization tool for policy makers in addressing the problem.

Rich 52 performed a scoping review of maternal health care in circumpolar regions and used a modified Delphi process to generate a core set of health system performance indicators.

### Outcomes achieved

The Conference enabled new networks to be established and existing ones consolidated. These cross sectors, disciplines, and national and regional boundaries. Knowledge users from territorial governments, regional health authorities and communities spoke at the conference, participated as panelists, and engaged in discussions at other sessions and informal social events. The Conference facilitated the transfer of research evidence to decision makers and providers for uptake and implementation to improve health systems serving remote communities.

## Footnotes

1 Bjerregaard P. Health reform in Greenland – Before and after

2 Mendez I. Narrowing the gap of inequality in healthcare delivery using remote presence technology

3 Hiratsuka V. Health outcomes from the NUKA system of care for Alaska Natives

4 Sigurðardóttir Á. Organization of health care in rural Iceland: Socioeconomic status, health related quality of life and urban–rural residence

5 Maguire G. Dynamics of health care access in vulnerable Aboriginal Australian communities

6 Lavoie J, Boyer Y, Kornelsen D. Circumpolar indigenous health policy: Lessons from a cross-national analysis.

7 Chatwood S. Social determinants and health systems stewardship.

8 Driscoll D. Looking upstream: Assessing the social and physical determinants of circumpolar population health to inform policy.

9 Johnson R. Viewing rural health through a health equity lens: Strategies and lessons learned.

10 Romain S, Kohler J, Young K. Pharmaceutical health care in Nunavut: The practice of policy from afar.

11 Drossos A. Mental health laws in Canada’s North: Do they help or hinder?

12 Andrews C, Kandola K, Brown S, Blake E. Healthy policies at the local government level: Building new partnerships in the Northwest Territories.

13 Rondeau K. Planning for health human resources in rural, remote and frontier First nations communities: A framework for action.

14 MacDonald WA, Berry J. Working together collaboratively: Community health nurses and family physicians in the Canadian North.

15 Jackman D, Yonge O, Konkin J, Janke F, Myrick F. Interprofessional preceptorship: Through the rural lens.

16 D’Hont T. Social accountability of the Canadian medical education system to the Northwest Territories.

17 The panel consists of 5 presentations: (1) Grongstad M, Norvik C, Norbye B, Wolff Skaalvik M. Decentralized nursing education in rural areas: A necessity in Northern Norway; (2) Norvik C, Grongstad M, Wolff Skaalvik M, Norbye B. The multifaceted role as a university lecturer in a decentralized nursing education program; (3) Edgecombe N, Robertson A. The Arctic Nursing Program: Building capacity in Canada’s Arctic; (4) Moffitt P. Decolonized nursing education at Aurora College; and (5) Butler L, Exner-Pirot H. Providing accessible nursing education in Yakutsk, Russia.

18 Moffitt P, Kulig J, Macleod M, Andrews ME, Stewart N, Ouellet M. Aboriginal rural and remote nurses in Canada.

19 Yue E. Exploring the extent of trust caregivers have in their community health workers in rural Uganda.

20 Montesanti S, Mah E, Berman J, Tobe S, Beardy J, Sodhi S. Launching a global network to facilitate multidirectional knowledge translation and exchange on inclusive and equitable non-communicable disease policy and practice for underserved populations.

21 Heffernan C, Komarnisky S, Nokohoo M, Piche D, Sack S, Sparling L, Long R. Pathways to health equity across jurisdictional boundaries: Building a team and crafting interventions.

22 Jardine C. Research partnerships to address health inequities in remote communities: The critical role of trust between academic researchers and indigenous communities.

23 Geary J, Highet M, Archie B, Carpenter B, Greenland W, Colquhuon A. A long-term collaborative research project to address community health concerns about a stomach infection: Perspectives from community and academic partners in the CANHelp Working Group.

24 Healey G. The Sangilirviuksaq Healing Model: Designing an Inuit healing centre in the Arctic that is responsive to how Inuit view the relationship between wellness and the land.

25 White M, Healey G, Cox G, Aspinall N, Pin G. Arctic adaptations: Sangilirviuksaq Healing Network.

26 Larcombe L, Coar L, Yassie E, Denechezhe L, Orr P, Kinew K, Singer M. Dene healthy housing in Manitoba: New approaches for building health equity.

27 Morar I, Caron-Roy A. Lots of space, nowhere to go.

28 Anderson W, Kandola K, Blackman F, Dobson B, Hartley-Folz S. Working on wellness; Workplace wellness to encourage healthy behavior in worksites of the Northwest Territories.

29 There were two linked presentations by Sawatzky A, Willox AC, Bunce A, Gillis D, Shiwak I, Shiwak J, Wood M, Ford J, Furgal C, Edge V, Harper S. (1) Inuit perspectives on community-based, participatory approaches for addressing health impacts of environmental change in Rigolet, Nunatsiavut, Labrador; (2) InukSUK (Strength, Understanding, Knowledge): A community-driven environment and health surveillance program in the Canadian Arctic.

30 There were two linked presentations by Clark D, Ford J, Pearce T, Berrang-Ford L. (1) Vulnerability to injuries associated with land-use activities in Nunavut, Canada; (2) The role of environmental factors in search and rescue incidents in Nunavut.

31 Young S, Blondin B. How we used to be: Challenges and lessons learned from implementing healing through wellness on the land.

32 MacKenzie J. Reclaiming our health and connections to the land by fostering youth leadership and talent: The role of participation action video research at Kalemi Dene School (Yellowknife).

33 MacDonald WA, Jong M, Bjerregaard P, Pontin D. Panel on medevac systems in circumpolar regions.

34 White M, Sheppard L. Next North: Health hangars.

35 Orkin A, Ritchie S, Mew E, Vanderburgh D. Community-based emergency care: Addressing the absence of emergency medical services in remote First Nations communities.

36 Sherman J, Wolff D, Prevost C, Nixon J, Ritchie S. Developing a sustainable model of community paramedicine for northeast Ontario: Lessons learned.

37 Jong M. Telehealth for remote communities.

38 Kawaguchi A, Sharafeldin N, Sundaram A, Campbell S, Rudnisky C, Weis E, Tennant M, Damji K. Tele-ophthalmology for age-related macular degeneration and diabetic retinopathy: A systematic review and meta-analysis.

39 Edin-Liljegren A, Holmner A, Lundqvist A, Berggren P. The Virtual Health Room: Bringing health service to the patient.

40 Ross A, Edie A, Yap T, Martin K, Van der Nest J. Increasing primary care access for residents of remote communities in Northern Alberta.

41 Ferrazzi P. Remote technologies for responding to people with mental illness in Nunavut criminal courts: A qualitative examination of current capacity and potential.

42 Manca D, Kandola K, Aubrey-Bassler K, Campbell-Scherer D, Moineddin R, Meaney C, Aguilar C, Socak N, Cullen R, Baxter J, Grunefeld E. Implementing a BETTER way to chronic disease prevention and screening in the Northwest Territories.

43 Joschko J, Liddy C, Afkham A, Keely E. Patient perspectives on electronic consultation as an acceptable alternative to traditional referrals: The Champlain BASE eConsult services.

44 Ryan S, Cranfield K, Naylor C, Swales K, Wilson W. How the Yukon Baby app came to be and early feedback.

45 McCready C. Improving the consultation process between community health nurses and physicians.

46 Lewis C, Lazurko M. Impact of home health monitoring on patients with heart failure.

47 Young K, Chatwood S, Marchildon G. Health care in Canada’s North: Are we getting value for money?

48 Galloway T, Duncan L, Hanley B, Lavoie J, Willows N, Macdonald D. Impact of federal health program funding models in Yukon First Nations.

49 Ashbolt N, Shoults D. Designing sanitation and water services within One Heath for remote Arctic communities.

50 Manca D, Forst B, Duerksen K. The Canadian Primary Care Sentinel Surveillance Network (CPCSSN): Transforming practice by bridging the evidence practice gap.

51 Moffitt P. Mapping rural and northern community response to intimate partner violence.

52 Rich R, D’Hont T, Linton J, Murphy K, Veillard J, Chatwood S. Selecting performance indicators for maternity care in a circumpolar context: A modified Delphi approach.

## References

[CR1] Chatwood S, Bytautas J, Darychuk A, Bjerregaard P, Brown A, Cole D, Hu H, Jong M, King M, Kvernmo S, Veillard J (2013). Approaching a collaborative research agenda for health systems performance in circumpolar regions. Int J Circumpolar Health.

[CR2] Chatwood S, Paulette F, Baker R, Eriksen A, Hansen KL, Eriksen H, Hiratsuka V, Lavoie J, Lou W, Mauro I, Orbinski J, Pabrum N, Retallack H, Brown A (2015). Approaching Etuaptmumk – introducing a consensus-based mixed method for health services research. Int J Circumpolar Health.

[CR3] Lavoie JG, Forget EL, Prakash T, Dahl M, Martens P, O’Neil JD (2010). Have investments in on-reserve health services and initiatives promoting community control improved First Nations’ health in Manitoba?. Soc Sci Med.

[CR4] Lavoie JG (2013). Policy silences: why Canada needs a national First Nations, Inuit and Metis health policy. Int J Circumpolar Health.

[CR5] Mendez I, Jong M, Keays-White D, Turner G (2013). The use of remote presence for health care delivery in a northern Inuit community: a feasibility study. Int J Circumpolar Health.

[CR6] Oosterveer TM, Young TK (2015). Primary health care accessibility challenges in remote indigenous communities in Canada’s North. Int J Circumpolar Health.

[CR7] Romain SJ (2013). Pharmaceutical health care and Inuit language communications in Nunavut. Can Int J Circumpolar Health.

[CR8] Romain SJ, Kohler JC, Young K (2015). Policy versus practice: a community-based qualitative study of the realities of pharmacy services in Nunavut, Canada. Int J Pharmaceut Policy Pract.

[CR9] Young SK, Tabish TB, Pollock NJ, Young TK (2016). Backcountry travel emergencies in Arctic Canada: a pilot study in public health surveillance. Int J Environ Res Public Health.

[CR10] Young TK, Chatwood S (2011). Health care in the North: what Canada can learn from its circumpolar neighbours. CMAJ.

[CR11] Young TK, Ng C, Chatwood S (2015). Assessing health care in Canada’s North: what can we learn from national and regional surveys?. Int J Circumpolar Health.

